# Effects of freezer storage time on levels of complement biomarkers

**DOI:** 10.1186/s13104-017-2885-1

**Published:** 2017-11-06

**Authors:** Angharad R. Morgan, Caroline O’Hagan, Samuel Touchard, Simon Lovestone, B. Paul Morgan

**Affiliations:** 1 Division of Infection and Immunity, School of Medicine, Cardiff University, Heath Park, CF14 4XN, Cardiff, UK; 2 Department of Psychiatry, University of Oxford, Oxford, UK

**Keywords:** Complement, Biomarker, Plasma, − 80 °C storage

## Abstract

**Background:**

There is uncertainty regarding how stable complement analytes are during long-term storage at – 80 °C. As part of our work program we have measured 17 complement biomarkers (C1q, C1 inhibitor, C3, C3a, iC3b, C4, C5, C9, FB, FD, FH, FI, TCC, Bb, sCR1, sCR2, Clusterin) and the benchmark inflammatory marker C-reactive protein (CRP) in a large set of plasma samples (n = 720) that had been collected, processed and subsequently stored at – 80 °C over a period of 6.6–10.6 years, prior to laboratory analysis. The biomarkers were measured using solid-phase enzyme immunoassays with a combination of multiplex assays using the MesoScale Discovery Platform and single-plex enzyme-linked immunosorbent assays (ELISAs). As part of a post hoc analysis of extrinsic factors (co-variables) affecting the analyses we investigated the impact of freezer storage time on the values obtained for each complement analyte.

**Results:**

With the exception of five analytes (C4, C9, sCR2, clusterin and CRP), storage time was significantly correlated with measured plasma concentrations. For ten analytes: C3, FI, FB, FD, C5, sCR1, C3a, iC3b, Bb and TCC, storage time was positively correlated with concentration and for three analytes: FH, C1q, and C1 inhibitor, storage time was negatively correlated with concentration.

**Conclusions:**

The results suggest that information on storage time should be regarded as an important co-variable and taken into consideration when analysing data to look for associations of complement biomarker levels and disease or other outcomes.

**Electronic supplementary material:**

The online version of this article (10.1186/s13104-017-2885-1) contains supplementary material, which is available to authorized users.

## Background

Complement is an important part of innate immunity and plays a key role in inflammatory processes. The complement pathway includes more than 30 component proteins, regulators and receptors, which work together to fight infection and to clear toxic material. Concentrations of complement biomarkers in plasma have been found to be altered in a wide range of different diseases, including cancer, atherosclerosis, diabetes, inflammatory bowel disease, neurodegenerative disorders, autoimmune diseases and infections [1].

Levels of complement biomarkers are often inconsistent between different laboratories due in part to a lack of standardisation with variation occurring in sample collection, handling and storage [2–4]. As part of our work program assessing biomarkers in dementia, we have measured 17 complement biomarkers (C1q, C1 inhibitor (C1inh), C3, C3a, iC3b, C4, C5, C9, FB, FD, FH, FI, TCC, Bb, sCR1, sCR2, clusterin) and the benchmark inflammatory marker C-reactive protein (CRP) in a large set of plasma samples (n = 720) that had been collected, processed and subsequently stored at – 80 °C over a period of 4 years. Before investigating any possible association with dementia the biomarker levels were assessed for relationships with relevant co-variables that could influence the main results. Here we report our investigation of the impact of the freezer storage time on the measurement of complement biomarkers.

## Methods

### Samples

Samples were from the AddNeuroMed study, a cross-European cohort for biomarker discovery. Informed consent was obtained for all subjects according to the Declaration of Helsinki (1991) and protocols and procedures were approved by the relevant Institutional Review Board at each collection site. Further information regarding the cohort has been previously described [5, 6]. This study utilised plasma samples from 720 subjects: 262 with Alzheimer’s disease, 199 with mild cognitive impairment, and 259 elderly controls with no dementia.

Plasma samples were collected into clinical grade EDTA tubes (K_2_EDTA; final concentration 1.8 g/l) and centrifuged at 3000 rpm for 8 min at 4 °C. Plasma was harvested, aliquoted and then frozen at – 80 °C.

### Measuring the levels of clusterin, soluble complement receptor 2 and CRP

The plasma levels of clusterin, soluble complement receptor 2 (sCR2) and CRP were determined using commercially available enzyme-linked immunosorbent assay (ELISA)—compatible antibody pairs (clusterin and CRP from R&D systems (Abingdon, UK) (cat# DY5874 and DY1707) and sCR2 from Sino Biological (Beijing, China) (cat# SEKA10811)) and protocols were followed as described by the manufacturers.

### Measuring the levels of soluble complement receptor 1, C1-inhibitor, C5, C9 and C1q

The plasma levels of soluble complement receptor 1 (sCR1), C1-inhibitor (C1inh), C5, C9 and C1q were determined using optimised antibody pairs in sandwich ELISAs developed in-house. See Table 1 for details of each assay. Further information regarding our in house ELISA methods has been previously described [7].

**Table 1:** In-house ELISAs

Analyte	Capture antibody	Detection antibody	Standard curve	Plasma dilution
sCR1	1 µg/ml RP anti-CR1	1 µg/ml MM HRP labelled anti-human CR1	50, 25, 12.5, 6.25, 3.125, 1.565, 0.78, 0 ng/ml	1:2
C5	1 µg/ml RP anti-C5	1 µg/ml MM HRP labelled anti-human C5	1000, 500, 250, 125, 62.5, 31.25, 15.625, 0 ng/ml	1:100
C9	1 µg/ml MM anti-C9	1 µg/ml RP HRP labelled anti-human C9	100, 50, 25, 12.5, 6.25, 3.125, 1.5625, 0 ng/ml	1:2000
C1q	2 µg/ml MM anti-C1q (commercial WL02 from Hycult)	1 µg/ml RP HRP labelled anti-human C1q	1000, 500, 250, 125, 62.5, 31.25, 15.625, 0 ng/ml	1:800
C1 inhibitor	1 µg/ml MM anti-C1 inhibitor	1 µg/ml RP HRP labelled anti-human C1 inhibitor	100, 50, 25, 12.5, 6.25, 3.125, 1.5625, 0 ng/ml	1:16,000

*MM* mouse monoclonal antibody, *RP* rabbit polyclonal antibody

### Measuring the levels of C3, C4, factor B, factor H, factor I, factor D, Bb, C3a, iC3b, and terminal complement complex

The majority of the complement proteins in this study were measured using customised v-plex assays from MesoScale Discovery (MSD; Rockville, Maryland, USA), using antibody pairs developed in-house. C3, C4, factor B (FB), factor H (FH) and factor I (FI) were analysed together in multiplex 1 and factor D (FD), Bb, C3a, iC3b and terminal complement complex (TCC) were analysed together in multiplex 2. All 8 analytes were measured by an electrochemiluminescence (ECL) immunoassay technique according to the manufacturer’s protocol. In brief, pre-coated plates were blocked with 150 μl/well 3% BSA in PBS at room temperature for 2 h with shaking at 600 rpm. Plasma samples were diluted 1:2000 for multiplex 1 and 1:2 for multiplex 2 in assay buffer (PBS containing 1% BSA and 10 mM EDTA) and 25 μl aliquots were added in duplicate to wells. A calibration curve comprising a series of fivefold dilutions of protein standard was run in duplicate on each plate. Plates were incubated with shaking at 600 rpm at room temperature for 60 min. After washing in PBS containing 0.01% Tween20, 25 μl of a mixture of the relevant SULFO-TAG-labelled detection antibodies diluted in assay buffer (1:100) was added and incubated with shaking at 600 rpm at room temperature for 60 min. After washing, 150 μl of 2 × MSD reading buffer was added to each well and ECL signal was measured on the Sector Imager 2400 (MSD).

### Qc

All standards and samples for all assays were tested in duplicate. The intra-assay and inter-assay coefficients of variation (CV) % were set at 25% and data for any sample with a CV above this was not included.

### Statistical analysis

Protein concentrations were determined automatically from standard curves plotted using GraphPadPrism5. The units of concentration for all analytes shown are nanogram per milliliter. Spearman correlation tests were used in GraphPadPrism5 to identify correlations between protein levels and time in freezer.

## Results

The concentrations of 18 complement biomarkers were measured in 720 plasma samples by solid-phase enzyme immunoassays. The means, ranges and standard deviations for each analyte are shown in Table 2. Results obtained are compatible with published values for these analytes, taking into account that this is a cohort of elderly individuals, many with significant pathology. A number of co-variables were considered in the analysis of the data, including length of storage of samples at – 80 °C. The time the samples had been stored in the freezer before the measurement of biomarkers ranged from 6.6 to 10.6 years (mean 9.1. SD 0.6). The samples had not undergone any previous freeze–thaws and were transferred from the collection centres frozen on dry ice.

**Table 2:** Means and range of protein levels (ng/ml)

Analyte	n	Min	Max	Mean	SD
C3	690	235,097	8423,462	1004,059	528,689
FI	701	7419	69,773	34,876	8314
FB	695	27,419	295,598	103,430	28,238
FD	719	1092	4695	2125	607
C5	720	13,405	47,415	26,369	5458
sCR1	715	4	38	12	4
C3a	703	38	339	109	46
iC3b	571	38	726	240	133
Bb	719	50	1325	128	81
TCC	717	32	2941	132	252
FH	674	69,782	630,889	252,816	72,781
C1q	720	33,250	363,624	177,275	44,275
C1 inhibitor	719	81,192	673,570	236,527	59,156
C4	702	32,722	719,480	174,190	78,337
C9	719	23,542	268,083	81,610	29,727
sCR2	712	46	625	175	70
Clusterin	720	112,694	532,722	226,729	42,704
CRP	702	70	11,864	1255	1422

The cohort was tested for correlations between individual biomarker levels and time in freezer (Table 3 and Additional file 1). With the exception of five analytes (C4, C9, sCR2, clusterin and CRP), storage time was significantly correlated with measured plasma value. For ten analytes: C3, FI, FB, FD, C5, sCR1, C3a, iC3b, Bb and TCC, storage time was positively correlated, samples yielding significantly higher concentrations with longer time in the freezer. Using a linear model it was determined that concentration was increased for each year a sample was stored in the freezer by 30.3% (304,216 ng/ml) for C3, 8.1% (2808 ng/ml) for FI, 5.2% (5413 ng/ml) for FB, 5.8% (123 ng/ml) for FD, 5.4% (1418 ng/ml) for C5, 4.4% (0.53 ng/ml) for sCR1, 12.1% (13 ng/ml) for C3a, 9.7% (23 ng/ml) for iC3b, 6.1% (8 ng/ml) for Bb and 12.3% (16 ng/ml) for TCC. The storage time was negatively correlated with three of the proteins: FH, C1q, and C1inh which all displayed statistically significant lower concentrations with longer time in the freezer. Using a linear model it was determined that concentration decrease for each year a sample was stored in the freezer was 4.5% (11,452 ng/ml) for FH, 7.5% (13,285 ng/ml) for C1q and 2.8% (6710 for ng/ml) for C1inh.

**Table 3:** Test for correlation between analytes and time in freezer

Analyte	Spearman r	95% CI	p
C3	0.36	0.29 to 0.42	< 0.0001
FI	0.22	0.14 to 0.29	< 0.0001
FB	0.14	0.06 to 0.21	0.0003
FD	0.13	0.06 to 0.21	0.0003
C5	0.14	0.07 to 0.21	0.0001
sCR1	0.09	0.02 to 0.17	0.0118
C3a	0.17	0.09 to 0.24	< 0.0001
iC3b	0.1	0.02 to 0.19	0.0142
Bb	0.15	0.07 to 0.22	< 0.0001
TCC	0.27	0.20 to 0.34	< 0.0001
FH	− 0.11	− 0.18 to − 0.03	0.0056
C1q	− 0.1	− 0.18 to − 0.03	0.0064
C1 inhibitor	− 0.09	− 0.16 to − 0.01	0.0197
C4	0.003	− 0.07 to 0.08	0.93
C9	− 0.05	− 0.13 to 0.02	0.17
sCR2	− 0.02	− 0.09 to 0.06	0.63
Clusterin	− 0.01	− 0.09 to 0.07	0.75
CRP	0.03	− 0.05 to 0.10	0.51

## Discussion

Many plasma samples are stored for a number of years in the freezer before their use in biomarker studies and there are questions regarding how stable complement analytes are during long-term storage at – 80 °C. As part of our work program we had measured a large panel of complement biomarker analytes in a relatively large plasma set that had been stored at – 80 °C for between 6.6 and 10.6 years. As part of a post hoc analysis of extrinsic factors (co-variables) affecting the analyses we investigated the impact of freezer storage time on the values obtained for each complement analyte. Of the 18 analytes measured, ten had increased levels with longer time of storage at − 80 °C, three had decreased levels and five were not significantly changed. Notably, all four of the complement activation products (C3a, iC3b, Bb, TCC) included in the analysis were strongly positively correlated with storage time—all increased significantly in measured level. This suggests that there is significant “activation” of complement in EDTA plasma on long-term storage even at − 80 °C.

The study we report here is not without its limitations, the main one being that only one sample per subject was available. The best approach may be to measure samples at the time of collection and periodically thereafter using aliquots of the same samples on the same assay platform. However, such an experimental set-up is logistically difficult and even this is not without its own limitations as when comparing measurements taken at different times it is difficult to control for the variability this introduces. Measurements taken years later will be taken with different reagent batches and more than likely by different personnel.

There are few reports on the impact of storage time on levels of individual complement analytes and with only very small sample sets. Mollnes and co-workers made a pool of EDTA plasma obtained from 40 healthy blood donors; they stored the plasma for 3 years at − 70 °C, and compared with a similar fresh pool. When examined together, these two pools showed exactly the same amount of C3 activation products (15 AU/ml) and TCC (5 AU/ml) [2]. In another study of storage of 88 plasma samples at − 80 °C for 0–6 years, no significant effect of length of storage time was found on levels of complement activation products C3a, C4d, C5a, TCC (C5b-9 in this study) and Bb [8]. Our samples had been stored for longer than those in the described studies and this might explain in part the differences between our observations and those in these published studies. More likely is that in our study, the power provided by the large sample set (720 samples), and number of analytes, reveals storage effects not apparent in the published smaller studies.

There are further reports on the impact of storage time on non-complement biomarkers. One study reported that a 4-year difference in long-term storage had minimal effect on protein in plasma [9] while three other studies did report changes in protein biomarker levels with storage time. One study performed a time course analysis of cytokines, chemokines and growth factors measured in the banked serum of healthy donors and melanoma patients stored for various intervals, and analysed by multiplex Luminex assays. Seven of the ten analytes examined showed highly significant changes during the approximately 5 years of storage at − 80 °C [10]. Enroth and co-workers found that plasma values of 18 of 108 protein biomarkers investigated were influenced by storage time (storage time 2–28 years) [11], while Kugler et al. found that levels of two serum markers measured at sample collection and again in the same samples after approximately 10 years in storage increased by 15% [12]. Taken together, these findings demonstrate that storage time needs to be a consideration in all biomarker studies using stored plasma samples, not just those focussing on complement.

If the effects of storage can be modelled then it may be possible to introduce a correction factor for time of storage that “normalises” the data and permits the use of samples stored for different times without compromising a study. When analysing our own data further we plan to adjust the complement concentrations observed based on linear models and estimate the concentration of each sample based on a time in freezer measure of 9 years, chosen because it represents the mean time of storage in the study, thus minimising the applied adjustment.

## Conclusions

The key finding of this study is that long-term storage of plasma even at − 80 °C alters levels of some complement components and all complement activation products. These effects must be taken into consideration when analysing data from historical sample sets spanning long periods of storage time, the case in most biobanks and other archived collections. Information on storage time must be regarded as an important co-variable, just as important as patient age or gender, and taken into consideration (and corrected for) when analysing data to look for associations of complement biomarker levels and disease or other outcomes.

## Additional file

**Additional file 1.** Scatterplots of the levels of complement biomarkers measured in plasma depending on freezer storage time

## Abbreviations

C1inh: C1-inhibitor
CRP: C-reactive protein
CV: coefficients of variation
FB: factor B
FH: factor H
FI: factor I
sCR1: soluble complement receptor 1
sCR2: soluble complement receptor 2
TCC: terminal complement complex
