# Supplementary material for: Effects of freezer storage time on levels of complement biomarkers
Source: BMC Res Notes. 2017 Nov 6;10:559. doi: 10.1186/s13104-017-2885-1 (PMC5674861; doi:10.1186/s13104-017-2885-1)
Supplement: Supplementary file 1 — Additional file 1. Scatterplots of the levels of complement biomarkers measured in plasma depending on freezer storage time [file 13104_2017_2885_MOESM1_ESM.docx]

**Additional file 1**

***Scatterplots of the levels of complement biomarkers measured in plasma depending on freezer storage time.***

*Years in freezer are on the x-axis and protein concentrations (ng/ml) on the y-axis.*

For ten analytes storage time was positively correlated, samples yielding significantly higher concentrations with longer time in the freezer:

C3 (spearman r = 0.36, p < 0.0001), FI (spearman r = 0.22, p < 0.0001), FB (spearman r = 0.14, p = 0.0003), FD (spearman r = 0.13, p =0.0003), C5 (spearman r = 0.14, p = 0.0001), sCR1 (spearman r = 0.09, p = 0.012), C3a (spearman r = 0.17, p < 0.0001), iC3b (spearman r = 0.10, p = 0.015), Bb (spearman r = 0.15, p < 0.0001) and TCC (spearman r = 0.27, p < 0.0001).

The storage time was negatively correlated with 3 of the proteins which all displayed statistically significant lower concentrations with longer time in the freezer:

FH (spearman r = -0.11, p = 0.0056), C1q (spearman r = -0.10, p = 0.0064), and C1 inhibitor (spearman r = -0.09, p = 0.02).

For five analytes concentration and time in freezer was not correlated: C4, C9, sCR2, clusterin and CRP.
